# Possible therapeutic targets for NLRP3 inflammasome-induced breast cancer

**DOI:** 10.1007/s12672-023-00701-7

**Published:** 2023-06-10

**Authors:** Xixi Wang, Junyi Lin, Zhe Wang, Zhi Li, Minghua Wang

**Affiliations:** 1 Department of General Surgery, Taihe Hospital, Hubei University of Medicine, Shiyan, 442000 China; 2 Sinopharm Dongfeng General Hospital, Hubei University of Medicine, Shiyan, 442000 China; 3 Hubei Key Laboratory of Wudang Local Chinese Medicine Research, Hubei University of Medicine, Shiyan, China; 4 Interventional Cancer Institute of Chinese Integrative Medicine, Putuo Hospital, Shanghai University of Traditional Chinese Medicine, Shanghai, 200333 China; 5 Hubei Clinical Research Center for Precise Diagnosis and Treatment of Liver Cancer, Taihe Hospital, Hubei University of Medicine, Shiyan, China

**Keywords:** Inflammasome, NLRP3, Breast cancer, Nanoparticles, Gene therapy

## Abstract

Inflammation plays a major role in the development and progression of breast cancer(BC). Proliferation, invasion, angiogenesis, and metastasis are all linked to inflammation and tumorigenesis. Furthermore, tumor microenvironment (TME) inflammation-mediated cytokine releases play a critical role in these processes. By recruiting caspase-1 through an adaptor apoptosis-related spot protein, inflammatory caspases are activated by the triggering of pattern recognition receptors on the surface of immune cells. Toll-like receptors, NOD-like receptors, and melanoma-like receptors are not triggered. It activates the proinflammatory cytokines interleukin (IL)-1β and IL-18 and is involved in different biological processes that exert their effects. The Nod-Like Receptor Protein 3 (NLRP3) inflammasome regulates inflammation by mediating the secretion of proinflammatory cytokines and interacting with other cellular compartments through the inflammasome's central role in innate immunity. NLRP3 inflammasome activation mechanisms have received much attention in recent years. Inflammatory diseases including enteritis, tumors, gout, neurodegenerative diseases, diabetes, and obesity are associated with abnormal activation of the NLRP3 inflammasome. Different cancer diseases have been linked to NLRP3 and its role in tumorigenesis may be the opposite. Tumors can be suppressed by it, as has been seen primarily in the context of colorectal cancer associated with colitis. However, cancers such as gastric and skin can also be promoted by it. The inflammasome NLRP3 is associated with breast cancer, but there are few specific reviews. This review focuses on the structure, biological characteristics and mechanism of inflammasome, the relationship between NLRP3 in breast cancer Non-Coding RNAs, MicroRNAs and breast cancer microenvironment, especially the role of NLRP3 in triple-negative breast cancer (TNBC). And the potential strategies of using NLRP3 inflammasome to target breast cancer, such as NLRP3-based nanoparticle technology and gene target therapy, are reviewed.

## Introduction

Breast cancer(BC) is the most common cancer in women, and it is one of the leading causes of death worldwide [1–3]. According to molecular and histological evidence, BC can be divided into three categories. In addition to hormone-receptor-positive breast cancer (ER + or PR +), there are triple-negative breast cancers (TNBC) that express receptors for estrogen, progesterone, and HER2 [4, 5]. The molecular characteristics of BC should determine treatment. Further classification of TNBCs is based on their basal-like characteristic and basal-like consistency. These categories include basal-like 1 (BL-1), basal-like 2 (BL-2), immunomodulatory (IM), mesenchymal (M), and mesenchymal stem cell-like (MSL) (Table 1).

**Table 1:** Breast cancer subtypes category

Breast cancer subtype	Receptor profile	Subtype prevalence (%)
Triple negative breast cancer	ER-,PR-and HER2-	10–20
HER2 positive	HER2 +	20
Hprmone positive	ER + or PR +	60

As targeted therapies are lacking, a need for new therapies arises, and considering the huge molecular heterogeneity of breast cancer, new therapeutic approaches are urgently needed. As molecular analysis techniques continue to advance, breast cancer biology can now be approached at multiple levels of the omics interaction network, including genomics, epigenomics, transcriptomics, proteomics, and metabolomics. Individual tumor molecular characteristics can be targeted in specific ways thanks to precision oncology [6]. Targeted therapies for breast cancer have traditionally only been based on hormone receptors (HR) and HER2 status: for patients with estrogen- and progesterone-positive (ER/PR +) cancer, endocrine therapy (e.g., tamoxifen) is used, whereas for patients with HER2 + cancer, antiHER2-targeted therapy is used [7]. As a successful marker for implementing immunotherapy (e.g., atezolizumab and pembrolizumab) in combination with chemotherapy (e.g., NAB-paclitaxel) for TNBC patients without HR or significant HER2 overexpression [8, 9], the expression of programmed death ligand 1 (PD-L1) has recently been identified. The biomarkers used in treatment decisions are still mainly HR and HER2, despite decades of research into the molecular characterization of breast cancer. Therefore, treatment strategies are still not sufficiently tailored.

The inflammasome is a multi-protein complex in cells, which plays a vital role in innate immunity by recognizing pathogens and various danger signals and inducing inflammatory responses [10]. The NOD-like receptor family, pyrin domain-containing protein 3 (NLRP3) inflammasome is one of the most well-defined inflammasomes. NLRP3 protein, Apoptosis associated speck-like protein, ASC), and caspase-1 precursor assemble to further recruit and promote self-activation of pro-caspase-1, which cleaves Interleukin 1 beta (Il-1β). IL-1β) and IL-18 (IL-18) precursors become active. These products can activate downstream signaling pathways, promote the release of inflammatory factors, and enhance the inflammatory response [11]. In the past decade, studies on the NLRP3 inflammasome mostly focused on the field of innate immunity. However, there were few studies on its mechanism of action and clinical significance in the occurrence and development of cancer. The conclusions of the research were not uniform [12]. Studies have shown that NLRP3 is not only expressed in innate immune cells, but also in epithelial cells [13], and is involved in the progression of some epithelial-derived tumors [14]. Breast cancer is typically a malignant tumor of epithelial origin. There are few reports on the expression and role of the NLRP3 inflammasome in breast cancer. This review article discusses NLRP3 and its role in breast cancer, including its potential as a therapeutic target.

## NLRP3

### NLRP3 structure

Among the greatest discoveries in contemporary immunology, pattern recognition receptors (PRRs) and their signal transduction in innate immunity have greatly influenced research in immunology. Toll-like receptors (TLRs), RIG-I-like receptors (RLRs), and NOD-like receptors (NLRs) have all been identified as PRRs. Cell-to-cell receptors called TLRs and NLRPs recognize molecules associated with pathogens. Aspects of damage-related molecular patterns (DAMPs) and process-associated molecular patterns (PAMPs) [15, 16].

**Fig. 1:**
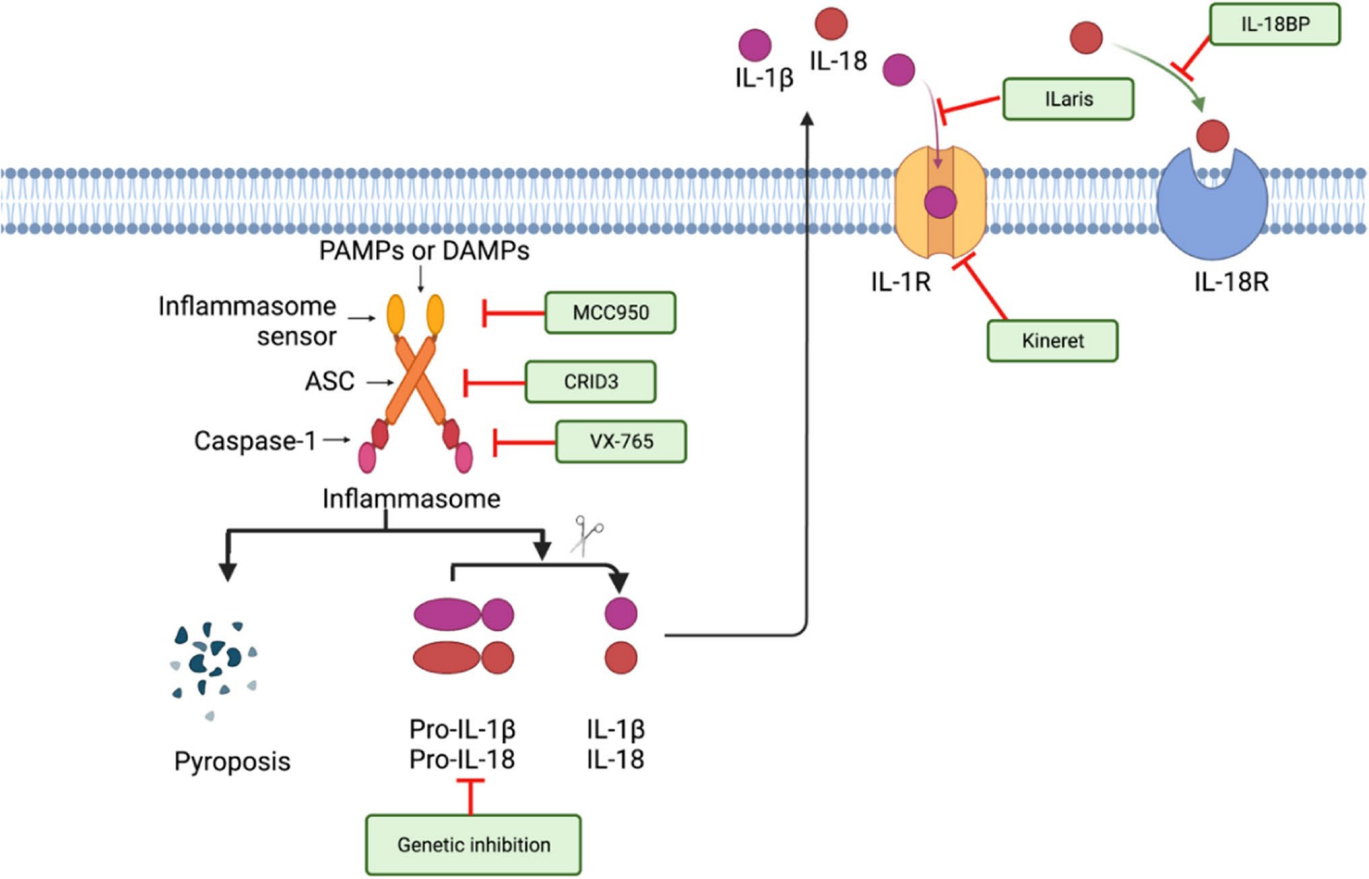
Therapeutic targets of the inflammasome pathway. Recognition of pathogen-associated molecular patterns (PAMPs) or danger-associated molecular patterns (DAMPs) by inflammasome-initiating sensors leads to the activation of the inflammasome and initiation of pyroptosis and release of the bioactive form of IL-1β and IL-18 [18]. IL-1β and IL-18 engage in autocrine and paracrine signaling pathways via the IL-1 receptor (IL-1R) and IL-18 receptor (IL-18R), respectively. The inflammasome signaling pathway can be inhibited by pharmacological inhibition of activation of the inflammasome (MCC950) [19], ASC oligomerization (CRID3) [20], caspase-1 (VX-765) [21], and IL-1R (Kineret) [22], or neutralizing IL-1β (Ilaris) [23] or IL-18 (IL-18BP) [24]

In addition to NLRP3, there are 22 human-derived proteins and at least 34 murine proteins in the NLR family. The NLR oligomerization is mediated by its nucleotide-binding oligomerization domain (NACHT). LRRs (leucine-rich repeats) at the C-terminus are involved in recognizing stimuli. Besides caspase recruitment domains (CARDs), the N-terminal effector domain contains two pyrin domains (PYDs) that mediate downstream protein interactions [17] (Fig. 1).

### Activation of NLRP3 inflammasome

NLRP3 consists of three domains: a carboxy-terminal leucine-rich repeat, a nucleotide-binding and oligomerization domain (NACHT) with ATPase activity, and an amino-terminal pyranan domain (PYD) [25]. NLRP3 inflammasomes are usually not activated by basal levels of its expression.

Consequently, two steps are required to initiate and activate the process. Inflammasome aggregation is induced by TLRs and cytokine receptors, such as tumor necrosis factor (TNF) receptors and interleukin-1 receptors (IL-1R), when these receptors recognize DAMPs or PAMPs and up-regulate NLRP3 and IL1B transcription. During this process, Caspase-1 (CASP1), a pro-inflammatory cytokine, and pyroptosis are activated [26]. Among these PAMPs and DAMPs are microbial activators, or fractions derived from microbes or hosts. These can be extracellular ATP, uric acid crystals, calcium phosphate dihydrates, cholesterol crystals, or glucose, for example. However, in human monocytes, the priming step alone is sufficient to mediate CASP1 activation and IL-1β release.Primary and metastatic mammary tumors are promoted by IL-1 signaling [27].

In these cells, lipopolysaccharide (LPS) triggers the release of endogenous ATP, which activates the P2X7 receptors and activates the NLRP3 inflammasome. Human monocytes are also activated by LPS to activate another NLRP3 inflammasome pathway. Among them, receptor-interacting serine/threonine protein kinase 1 (RIPK1), Fas-associated death domain protein (FADD), and CASP8 are required for TLR4-Toll or interleukin-1 receptor domain-containing adaptors to become active downstream of interferon-β (TRIF) signaling [28]. Human and mouse NLRP3 activation differ slightly, but further studies are needed to fully understand them.

The mechanism of how the NLRP3 inflammasome aggregates are activated has been proposed to involve disruptions in cellular homeostasis as a result of multiple triggers. Apoptosis-related speck-like proteins containing CARD(ASC) are recruited by NLRP3 through interaction with ASC through interaction with its PYD domain [29]. The NLRP3-ASC-pro-CASP1 complex is formed by the CARD domains of ASC and pro-CASP1. As a result, pro-CASP1 is cleaved into active CASP1, which then cleaves IL-1β and IL-18 into their biologically active forms. (Fig. 1).

Antigen D (GSDMD) is also lysed by CASP1, causing plasma membrane pores through which inflammasomes with biologically active forms of cytokines (IL-1) are released, resulting in pyroptosis, an inflammatory form of cell death [30]. A second form of NLRP3 activation occurs during influenza A virus infection (IAV). NLRP3 inflammasomes, called ZBP1-NLRP3 inflammasomes, are required for pyroptosis and CASP1 cleavage by Z-DNA-binding protein 1(ZBP1). Inflammasomes consisting of ZBP1 and NLRP3 aggregate after ZBP1 detects Z-RNA during IAV infection by recruiting receptor-interacting serine/threonine protein kinase 3 (RIPK3) and CASP8 [31]. Several host proteins regulate this aggregation of inflammasomes following infection with IAV, including interferon signaling molecules and CASP6. As well as CASP11 or its human homologs CASP4 and CASP5, mouse CASP11 can activate NLRP3 through a noncanonical pathway [32]. Inflammasome activation requires pore formation for NLRP3 to recognize intracellular LPS and clear GSDMD.

NLRP3 inflammasome activation is tightly regulated by innate immune molecules during infection and inflammation. The NLRP3 inflammasome is activated and formed by a number of positive regulators. As a result of myeloid differentiation primary response 88 and TRIF, the NLRP3 inflammasome can activate. NLRP3 inflammasomes are also initiated and activated by CASP8 and FADD. Furthermore, DDX3X promotes the activation of NLRP3 by interacting with it [33]. The activation of the ZBP1-NLRP3 inflammasome during IAV infection is also promoted by CASP6 [31]. Inflammasome activation is also regulated by other upstream regulators, such as SHARPIN, immune-related GTPases, and interferon regulators.

Inflammation and hyperactivity of the NLRP3 inflammasome can also be prevented by negative regulators of the inflammasome. According to studies, E3 ligase A20/TNF-induced protein 3 inhibits inflammation in macrophages and in a mouse model of inflammatory arthritis, as well as negatively regulating NLRP3 inflammasomes. In tumor suppressor pathways, transcription factor p53 is one of the key players in the response to DNA damage, and an increasing number of target genes are known to play a part in these pathways. The active form of Resveratrol (RSV) inhibits NLRP3-mediated inflammasome activation by Sirt1 (SIRT1) / p53-dependent cellular aging. Moreover, TAK1 acts negatively to maintain NLRP3 inflammasome quietness and maintains cellular homeostasis.

## The role of NLRP3 in breast cancer

Malignant tumors in women are most commonly breast cancer. Inflammasomes are protein signaling complexes that release proinflammatory cytokines when stimulated by DAMPS and PAMPS. A number of studies have suggested that NLRP3 activation plays a role in the development of breast cancer [34, 35].

The activation of an inflammasome in the NLRP3 is one of the most important mechanisms through which tumor growth and metastasis are promoted in BC [36], which leads to tumor proliferation, angiogenesis, invasion, progression, and recurrence [37, 38]. The relationship between local levels of IL-1β and breast malignancy has been observed in BC mouse models [39]. As a result of CASP-1 activation, matrix metalloproteinase 9 (MMP-9) is produced, which plays a role in the progression of radioresistant BC to metastatic disease [40]. It has also been shown that NLRP3 decreases antitumor immunity in T cells and NK cells, which creates an inflammatory environment supporting BC progression and metastasis by activating inflammatory signaling pathways, such as the NF-κB/STAT 1/3 and IL-1β/IL-1RI/β-catenin pathways [36, 41].

Recent research has demonstrated that NLRP3 contributes to leptin-induced BC. An adipocyte-secreted hormone, leptin, promotes BC cell migration by activating the NLRP3 inflammasome and increasing IL-18 production [42, 43]. Growth of BC cells is also increased by activating the inflammasome. BC cell growth is inhibited by globular adiponectin by inhibiting the NLRP3 inflammasome [44]. NLRP3 inflammasome activation and mitochondrial function were all affected by breast cancer susceptibility gene(BRCA1) deficiency in an in vitro/in vivo study [45]. Inflammasome inhibition has been found to be a therapeutic target for BC based on the results of these studies.

### The role of NLRP3 in TNBC

Breast cancer, especially triple-negative breast cancers (TNBC), has a high cell proliferation rate and is prone to recurrence [46]. Martinon et al. discovered the inflammasome in 2002 to link inflammation, tumorigenesis, angiogenesis, and metastasis [47]. There are three components to the inflammasome complex: the sensor molecule, the adaptor protein, and the receptor (Fig. 1). PLRs, ALRs, RLRs, CLRs, and proteins with tripartite motifs, such as pyrin (also known as marenostrin or TRIM20) are among the molecules involved in the inflammasome [47].

A signal for activating the inflammasome is primed, and another signal for activating it is activation. As a result of priming stimuli from the inflammasome, TLRs are activated, FAS-associated death domain protein and IL-1 receptor ligands are activated and the NF-kB pathway is triggered, resulting in the up-regulation of the inflammasome. Normal conditions do not permit inflammasome activation due to low inflammasome components. Inflammasome regulation and pro-IL-1B secretion are mediated by two NF-kB pathway signaling molecules, MyD88 and TIR-domain-containing adapter-induced interferon-B (TRIF), but ASC, caspase-1, and IL-18 expression remain untouched. ASC oligomerization, inflammasome assembly, and caspase-1 activation are required following the priming signal induced by DAMPs or PAMPs [48]. As well as detecting the oligomerization of ASC, these sensors can also detect the activation of cysteine protease CASP1 [49]. The ASC adapter proteins form speck-like structures after specific agonists are detected by the receptors, and these structures recruit pro-CASP1 for cleavage into mature CASP1. CASP1 cleaves pro-inflammatory cytokines (IL-13 and IL-18) and pore-forming gasdermin D (GSDMD) to cause CASP1-dependent cell death, called pyroptosis, which is controlled by the gasdermin family (GSDMA, GSDMB, GSDMC, GSDMD, GSDME, and DFNB59) [50]. Based on the structure of the receptor triggering the signaling pathway, the inflammasome is classified, so this complex is divided into canonical inflammasome-forming NLRs (NLRP1, NLRP2, NLRP3, NLRP6, NLRP7, NLRP9) and NOD-like receptors C4 (NLRC4) and NLRC5), as well as non-canonical inflammasome-forming AIM2 (AIM2 and IFI16) and inflammasome-forming pyrin (MEFV) [51]. While several molecular aspects of the NLRP3 inflammasome have yet to be explored, it is the most studied in the BC context. Inflammasomes that do not involve CASP1 activity, such as human caspase-4/5 and murine caspase-11, are called non-canonical inflammasomes. Furthermore, it is still unknown what molecular mechanisms underlie non-canonical signaling [52].

Inflammasome NLRP3 activation, pro-inflammatory cytokine production, and pyroptotic death of cancer cells promote breast tumor growth, progression, and aggressiveness. The overexpression of NLRP3, IL-1β, IL-18, and CASP1 has been associated with inflammatory signaling pathways that are active during inflammation. The release of reactive oxygen species (ROS), reactive nitrogen species (RNS), angiogenic factors, cytokines, and chemokines is a characteristic of invasive TME, characterized by the release of reactive oxygen species (ROS), reactive nitrogen species (RNS), cytokines, and chemokines [53].

Pyroptosis occurs when cells become swollen, lysed, nucleated, and fragmented. Protumorigenic and antitumorigenic roles can be played by pyroptosis. NLRP3 and ASC oligomerization mechanisms are associated with the pro-tumorigenic role of pyroptosis, while immunogenic signals and immune responses are associated with its anti-tumorigenic role [54].

GSDMD undergoes proteolytic cleavage following NLRP3 activation and maturation of CASP1. GSDMD-derived pores of 10–20 nm in diameter attach to the cell membrane and allow calcium or sodium ions to enter and potassium ions to exit, thus stimulating the inflammatory process [55].

Recent years have seen an increase in research on the contribution of inflammasome complexes to various diseases. Cancer, obesity, and diabetes are some of these diseases. Inflammasomes play a dynamic role within the immune system and inflammasomes overlap with inflammatory mechanisms. A direct connection exists between inflammasome complexes and the immune system through the secretion and maturation of pro-inflammatory IL-1β. It induces tumor development by activating the NF-kB pathway and angiogenic signals [56].

TNBC is associated with the inflammasome, and numerous studies have investigated inflammasome components as potential anticancer strategies. The antitumor properties of the NLRP3 inhibitors (MCC950 and AcYVAD-cmk) or the identification of ncRNA molecules that are known to control transcriptional activity can all be achieved through further research on these potential inhibitors. The IL-1 signaling pathway can be inhibited by drugs such as anakinra, canakinumab, and rilonacept. Inhibitors of apoptosis have been shown to reduce tumor cell proliferation and initiate apoptosis [57]. A thorough study of the inflammasome is necessary to uncover therapeutic possibilities for TNBC. It is likely that more NLRP3 inhibitors will be developed in the future since several are currently in pre-clinical trials (Table 2).

**Table 2:** NLRP3 inhibitors in selected clinical studies

Drug	Product	Target	Status in USA	Indication	NCT identification number
Dapansutrile(OLT1177)	Olatec	NLRP3	Clinical phase II	Osteoarthritis, Acute gout attacks, Heart failure	NCT03595371 NCT02104050 NCT01768975 NCT03534297
Somalix	Inflazome	NLRP3	Clinical phase II	Parkinson's disease, Osteoarthritis, memory Coronary heart disease, Congestive heart failure, Coronary artery disease	NCT04015076 NCT04338997 NCT04086602
IFM-2427 (DFV890)	IFM Tre/Novartis	NLRP3	Clinical phase I	Crohn's disease, Coronary Artery disease, Gout, NAFLD	–
NT-0167	Nodthera	NLRP3	Clinical phase I	Hepatic fibrosis, Pulmonary Fibrosis, NAFLD	–
Undisclosed	Jecure/Genetech	NLRP3	Preclinical	Liver fibrosis, NASH	–
Inzomelid	Inflazome	NLRP3	Clinical phase II	CAPS	–

### The role of NLRP3 in Breast tumor microenvironment

Activation of inflammasomes promotes epithelial-mesenchymal transition (EMT), metastasis, and angiogenesis, inhibits apoptosis, and facilitates tumor progression. Furthermore, the inflammasome pathway is also connected with immune responses and pyroptosis, programmed death of tumor cells [58].

Inflammatory infiltrates are a prognostic marker of cancer in the breast tumor microenvironment (TME). Because pro-inflammatory molecules and their specific receptors can promote cell differentiation and angiogenesis, recruit immune cells, and influence the immune system, they play a role in the development of TNBC [59]. Among the common cell types in the TME, lymphocytes, macrophages, and fibroblasts secrete several factors (cytokines, chemokines, enzymes, growth factors, etc.), which contribute to an aggressive malignancy with high metastasis risks. There are many pro-inflammatory factors involved in inflammation, TNBC, invasion, and metastasis, including IL-1, IL-6, IL-8, IL-11, IL-18, and IL-23, which are generally up-regulated in breast tumor stroma [60]. Activation of the NLRP3 inflammasome complex is associated with the development of inflammatory reactions and TNBC to promote the proliferation and invasion of tumor cells [61]. As well as encouraging myeloid cells, especially MDSC, and TAMs, to enter the TME, NLRP3 is implicated in tumor lymphangiogenesis and lung metastasis [62].

Breast TME has developed new signaling pathways in recent years, which have opened up new opportunities for developing strategies to inhibit those signals that promote tumorigenesis, angiogenesis, and metastasis. A combination of anthracyclines and taxanes, capecitabine and taxane, and ixabepilone monotherapy is the gold standard for neoadjuvant treatment. There is adjuvant treatment (anthracycline-based drugs), surgery (mastectomy and lumpectomy), radiotherapy, chemotherapy, and paclitaxel as well as doxorubicin, cisplatin, carboplatin, abraxane, bevacizumab, cyclophosphamide, ixabepilone, capecitabine, etc. [63]. Although TNBC is heterogeneous and aggressive, neoadjuvant chemotherapy follows surgery and adjuvant chemotherapy (Table 2). It is common to administer anthracyclines and taxans for early-diagnose TNBC, but these drugs cause numerous side effects (nausea, fatigue, gastrointestinal toxicity, myelosuppression, alopecia, hypothyroidism, hyperthyroidism, pneumonitis, skin reactions, adrenal insufficiency, peripheral neuropathy, neutropenia, pyrexia, anemia, thrombocytopenia, electrolyte abnormalities, infection, etc.) and do not decrease the relapse rate [64].

TNBC tumors have molecular characteristics (heterogeneity and chemoresistance) that make traditional therapy difficult; therefore advanced methods have been developed. Advanced therapeutics are divided into passive and active transport (miRNA and aptamers). Passive transport (or enhanced permeability and retention (nanoparticles)) is a technique that improves molecules' ability to pass through cells [63].

Inflammasome signaling is closely correlated with immunosuppressive TMEs in breast malignancies. Immune signaling (cytokines, chemokines, T-cell receptors, etc.) activates NOD-like receptors, which activate inflammasomes. Further, immune pathways (such as TLRs) stimulate DAMP detection and induce intracellular signals that bring about ASC adaptor protein recruitment and inflammasome oligomerization upon activation [65, 66]. Further, HER2-targeted treatment is less effective in patients upregulated for gasdermin B (GSDMB) [67].

### Non-Coding RNAs in NLRP3 inflammasome and breast cancer

RNA molecules are copied from noncoding sequences by technological advances in sequencing, which can help regulate gene expression, cell growth, and organ development, as well as play a part in the development of a variety of human diseases, including cancer, autoimmune disorders, metabolic disorders, and inflammation. The role of ncRNAs in cancer is illustrated by mutations or aberrations in their promoters as well as dysregulation of enzymes involved in their biogenesis (Drosha, Dicer), which can alter gene expression levels, which are crucial to the body's normal functioning. In addition, ncRNA expression is associated with cancer progression and metastasis [68].

A number of oncomiRs have been studied, including miR-373, miR-135b, miR-210, miR-638, miR301b, and miR-663a. Initially identified as a miRNA involved in tumor-cell proliferation, migration, invasion, and metastasis via CD44 targeting, miR-373 was identified as a metastasis-promoting miRNA in breast cancer [69]. It is miR-373 that regulates the secretion and maturation of caspase-8, which is principally responsible for activating the inflammasome complex non-canonically [70]. Cell proliferation and invasion are enhanced via MiR-135b [71], while inflammation and CASP1 expression are inhibited [72]. Additionally, MiR-210 regulates hypoxia, programmed cell death, and tumor proliferation and metastasis through its functions in breast cancer biology [73]. MiR-301 is up-regulated in TNBC due to aberrant miR expression, and it promotes tumor growth, cell proliferation, migration, invasion, and tamoxifen resistance through the TLR4/NFkB signaling pathway [74]. A recent study revealed that miR-663a promotes apoptosis through the secretion and maturation of pro-inflammatory cytokines and is frequently deregulated in TNBC [75, 76]. Several studies have found that miR-638 activation modulates the ATP synthesis mechanism, contributing to the progression of TNBC and inflammasome assembly [77]. Moreover, miR-223 is a tumor suppressor that inhibits inflammation in TNBC. Research has demonstrated an association between miRNA-223 expression and NLRP3 inflammation inhibition [78, 79].

### MicroRNAs interplay with TNBC and NLRP3

Gene expression is influenced by miRNA molecules, which target and interfere with mRNA translation. miRNAs play a role in multiple biological functions, including cell growth, proliferation, migration, programmed cell death, and immune response. Several miRNAs are abnormally expressed in BC (especially TNBC) which promotes its progression.

Inflammasome modulation in breast TME is directly mediated by miRNAs, and it is also indirectly mediated by miRNAs modulating other signaling pathways, such as mTOR, MAPK, cAMP/PKA, AMPK, or IFN signaling [80]. Recent studies have also found that miRNAs are capable of regulating pyroptosis by activating or suppressing various molecular networks [81]. Several research groups conducted relevant studies that contributed to understanding how miRNAs regulate the assembly of inflammasomes and the activation of pyroptosis [82].

In accordance with Wang et al., miR-200b influences NLRP3, ASC, GSDMD, CASP1, IL-1β, and IL-18 expression. Based on their results, miR-200b up-regulation regulates the JAZF1/NF-kB signaling pathway, which is responsible for pyroptosis activation. NF-kB signaling is mediated by TGF-activated kinase 1 (TAK1), which is inhibited by JAZF zinc finger 1 (JAZF1). MiR-200b also had a limited effect on pyroptosis when JAZF1 expression was high [83].

TNBC also exhibits up-regulation of miR-155-5p. Xu et al. investigated in vitro how miR-155-5p antagomir enhances cetuximab's cytotoxic effects. Cetuximab and miR-155-5p antagomir stimulate pyroptotic cell death and decrease tumor growth through their interaction with GSMDE and CASP1. TNBC cells exposed to cetuximab were also shown to undergo pyroptosis and apoptosis when miR-155-5p was down-regulated [84].

Several miRNAs that are associated with the NLR family are implicated in inflammasome-mediated breast tumorigenesis and consequently influence the probability of TNBC. The miRs-125, 146b, 200, 223-3p, 373, 520, or 548 regulate NLRP3, NLRP1 and NLRP8 [85, 86]. MiR-143-5p and miR-210 regulate NLRP5, and miR-181 regulates NLRP8 [87]. The modulation of the inflammasome by these miRNAs affects TNBC through different signaling pathways, such as NF-κB, TGF-β or IL-6, which are also affected by the same miRNAs (miR-146b, miR-520, miR-373, etc.) and inhibit TNBC cell growth and proliferation [88, 89]. Several genes and mechanisms are regulated by miRNAs to influence the expression of the inflammasome and pyroptosis-related TNBC. Inflammasomes and pyroptosis are both involved in TNBC, although some studies have shown miRNA-associated networks.

MiRNA regulation overexpresses pro-inflammatory markers in NLRP3 components. Inhibiting the translation mechanism of NLRP3 mRNA and altering the release and maturation of pro-inflammatory cytokines are among the functions of MiR-223, a non-coding molecule and NLRP3 regulator [90]. The tumor suppressor miR-22 is another microRNA involved in NLRP3 regulation. MiR-22 expression correlates with difficulties in NLRP3 assembly, decreased cell proliferation rate, and low levels of metalloproteinase 2 (MMP2), MMP9, vimentin, and N-cadherin [91]. NLRP3 assembly is inhibited by miR-21 activities that regulate NLRP3 phosphorylation and lysine 63-ubiquitination and alter the normal functions of the immune system [92].

The microRNA miR-9 regulates the NLRP3 inflammasome. Furthermore, miR-9 has been shown to play an important role in tumor initiation, inflammation, and immunity regulation [93]. NF-kB signaling causes MiR-9 to inhibit the activation of the NLPR3 inflammasome, therefore inhibiting CASP1 expression and pro-inflammatory immune factors release (IL-1β and IL-18).

Given the critical role microRNAs play in TNBC progression and their ability to modulate NLRP3 components' expression, miRNAs may be effective therapeutic targets in TNBC treatment.

An RNA triple-helix structure composed of a miR-205 mimic and antagomiR-221 was developed by Conde's research team in 2016 to facilitate the evaluation of local anti-cancer therapies based on both oncomiR inhibition and tumor suppressor miR replacement. Upon miR delivery, data revealed that miR-205 and miR-221 stimulated cell adhesion, proliferation, migration, and metastasis in both in vitro and in vivo conditions by targeting laminin subunit gamma 1 (LAMC1). In contrast, miR-205 upregulation was caused by an increase in the p53 profile. Interestingly, miR-221 and miR-205 inhibition reduced E-cadherin, Slug, and Snail expression, resulting in reduced migration and invasion of TNBC cells. Additionally, this new strategy suppressed angiogenesis in TNBC contexts by lowering VEGF levels [94]. In addition to supporting breast tumor growth, MiR-205 inhibits apoptosis through its role in NLRP3 complex assembly. Under the control of miR-205, the expression of NLRP3, ASC, and CASP1 increases, triggering an inflammatory reaction that activates NLRP3 [95]. MiR-221 modulates breast tumorigenesis and inflammation [96]. Due to the reduction in Bcl-2 expression and suppression of the NLRP3/ASC/CASP1 signaling pathway, upregulation of miR-221 in TNBC has antiinflammatory effects and suppresses oxidative stress and programmed cell death [97].

A number of miRNAs have been intensively studied over the years in order to understand their role in TNBC progression, with the results of these studies showing that numerous miRNAs are involved in modulating the inflammasome complex, including miR-223-3p, miR-7-5p, miR-22-3p, miR-33, miR-9, miR-155. They interfere with translation by binding the 3'UTR of NLRP3 mRNA and binding conserved regions [98]. Xie et al. conducted a study examining miR-33's influence on NLRP3 inflammasome activation. There is no doubt that miR-33 is capable of attenuating mitochondrial oxygen consumption and increasing the production of reactive oxygen species in cells since it is responsible for the secretion of IL-1β and the increased expression of NLRP3 and caspase-1 in macrophages[99]. In contrast, Zhang et al. examined whether miR-233 might be used to inhibit inflammasome activity and the growth of breast tumor cells implicitly. NLRP3 activation inhibition caused by miR-233 inhibits tumor cell growth, migration, and invasion [100]. Based on the NLRP3 inflammatory pathway, Hu et al. reported that miR-155 plays a role in the aggressiveness of TNBC. As well as decreasing proliferative and colony-forming abilities of MDA-MB-231 cells, miR-155 interference increased apoptosis in tumor cells and inhibited pro-inflammatory cytokines production [101].

## NLRP3 emerging target therapy

### NLRP3 based nanotechnology

A number of studies indicate that NLRP3 inflammasome signaling is associated with carcinogenesis [102], and that inhibition of NLRP3 inflammasome can act as a cancer prevention strategy.Cancer medicine is entering a new era with nanotechnology-based therapies, and polymeric nanoparticles have become increasingly popular. Biological macromolecules, hydrophobic drugs, nucleic acids, and hydrophobic drugs can be loaded into it for localized, sustained drug release [103]. Nanoparticles interact with the immune system and are captured by cells that present antigens because of their pathogen-like size [104]. The immune system can be activated by nanoparticles using this property. A foreign nanoparticle may be recognized by the immune system and trigger an immune response in cancer immunotherapy [105]. The immune system responds to nanoparticles by secreting proinflammatory cytokines, which are a manifestation of an inflammatory response. Mice injected with cationic protamine DNA liposomes secrete proinflammatory cytokines, including TNF-α, IL-12, and IFN-γ [106]. The NLRP3 inflammasome is a major mediator of inflammatory responses [107]. Activation of the NLRP3 inflammasome leads to IL-1β secretion [108].

The polyethylenimine 25 kD molecule has high transfection efficiency and cytotoxicity. PEI-cyd (PC), previously synthesized using low-molecular-weight PEI (MW 600), is a substance which has shown a lower cytotoxicity than PEI 25 kD. The serum and liver showed increased levels of proinflammatory cytokines and spleen weight. A higher level of immune stimulation was induced by PEI 25kD/pDNA exposure than by PC/pDNA exposure. In addition, PEI 25 kD administration can cause NLRP3 inflammasome activation in breast cancer tissues and induce high levels of oxidative stress. According to these findings, polymeric nanocarriers may stimulate the immune system and promote cancer metastasis, which may affect the effectiveness of the cancer therapy.

An e-As4S4 formulation was applied to mouse breast cancer cells and tumor-bearing mice to overcome the problem of poor solubility of original As4S4. A bioavailable form of arsenic sulfide, e-As4S4, is being tested as a treatment for myeloid leukemia [109]. The effectiveness of hydrophilic arsenic sulfide nanoparticles (e-As4S4) has been evaluated in an animal model of highly metastatic triple-negative breast cancer (TNBC) using hydrophilic arsenic sulfide nanoparticles (e-As4S4). e-As4S4 significantly inhibited tumor metastasis to the lung and liver by eliminating NLRP3 inflammasomes in tumor tissues. To a certain extent, e-As4S4 could alter the inflammatory microenvironment in tumor tissues, thus prolonging the survival time of breast cancer mice [110]. The inflammasomes activated by ROS are also important in tumor-related inflammation [111]. In the study, it was shown that oral administration of e-As4S4 significantly increased arsenic accumulation and eliminated ROS from tumor tissues and reduced angiogenesis, inflammasome in the tumor microenvironment by inhibiting the expression of inducible factor-1α (HIF-1α), thereby inhibiting NLRP3 expression. In the r-As4S4 treatment group, arsenic hardly accumulated in tumor tissues. E-As4S4 depletes ROS and is cytotoxic, resulting in a less aggressive microenvironment for tumor cells. In addition, e-As4S4 inhibited the activation of hypoxia-inducible factor-1α and NLRP3 inflammasomes. Tumor-bearing mice live longer when angiogenesis is reduced, metastases are inhibited, and survival is prolonged. Since e-As4S4 can correct the microenvironment that leads to breast cancer, it has great potential as a treatment for this disease [112].

According to Zhu et al., Au4.5 nanoparticles can be used as a vaccine adjuvant by triggering NLRP3 inflammation and increasing antibody production by mediating caspase-1 maturation [113]. Based on these studies, the NLRP3 inflammasome appears to have therapeutic potential and can predict immunotherapy response.

According to Sadia [114], a viral spike tumor-activating pyroptosis agent (VTPA) is used for cancer-specific treatment of tumor pyroptosis induced by dual lysosomal disruptions and ROS generation. When VTPA is administered systemically, it can easily accumulate inside tumor cells and induce lysosomal disruption after containing organosilica-coated iron oxide nanoparticles and manganese dioxide protrusions. NLRP3 inflammasome is further activated by the degradation of manganese ions and iron oxide nanoparticles (IONPs) caused by the release of intracellular glutathione (GSH) overexpressed by tumors [115]. Tumor cells were activated by NLRP3 inflammasomes and released lactate dehydrogenase after VTPA treatment. First demonstration in vivo of cancer-specific pyrophosphorylation by structurally dependent and GSH-activated photoapoptotic agents provides a new paradigm for developing cancer-specific pyrophosphorylation nanodrugs.

### Nlrp3-Based gene therapy

Current therapeutic approaches targeting the NLRP3 inflammasome, including inhibitors of NLRP3 inflammasome upstream signaling or biological agents targeting IL-1β or its receptor, either indirectly inhibit NLRP3 or only partially inhibit NLRP3 inflammasome signaling, which limits their therapeutic efficacy [116, 117]. Genomic editing tools such as CRISPR/Cas9 are highly efficient, specific, and simple, making them an excellent option for directly targeting NLRP3 and removing inflammation caused by NLRP3.

Many inflammatory diseases can be treated by targeting NLRP3 inflammasomes, but facilitating current therapies remains a challenge. Through CRISPR/Cas9 gene editing, NLRP3 can be eliminated directly at the genomic level [118]. A study by Xu et al. [119] examined the delivery of Cas9 mRNA and RNA into macrophages using cationic lipid-assisted nanoparticles (CLANs). In order to inhibit NLRP3 activation in macrophages, mCas9 and gRNA encapsulated in mCas9 and gRNA target NLRP3 CLAN (gNLRP3 / CLANmCas9) using encapsulation techniques [120, 121]. CLANmCas9 / gNLRP3 alleviated acute inflammation following intravenous administration in patients with LPS-induced septic shock and MSU-induced peritonitis. CRISPR/Cas9 can be delivered to macrophages via a vector developed in this study to treat NLRP3-dependent inflammatory diseases. Breast cancer may be treated with new ideas by targeting the NLRP3 inflammasome through gene editing.

Upon encountering a pathogen, inflammasomes accumulate in the cytoplasm, triggering caspase-1-dependent maturation of proinflammatory cytokines and pyrophosphorylation cell death. At the same time, NLRP3 is associated with many sterile inflammatory diseases because of its role in pathogen defense. Many common human diseases, such as atherosclerosis, type 2 diabetes, Alzheimer's disease, or gout, have been linked to NLRP3 inflammasomes. The molecular mechanism of NLRP3 activation remains elusive, despite the fact that known NLRP3 stimuli activate potassium efflux upstream of NLRP3 [122]. The activation of NLRP3 inflammasomes requires also NEK7, a serine/threonine kinase. Structures of NEK7 and NLRP3 determined by cryo-electron microscopy suggest that NEK7 binds to NLRP3's nucleotide-binding domain and leucine-rich repeat. In a flow cytometry-based NLRP3-dependent cell death screen using nigericin as a potassium efflux stimulator, Jonathan L et al. [123] found that targeting Nek7 saved macrophages from nigromycin-induced death. In contrast, AIM2-mediated activation of the inflammasome was not affected by Nek7 loss in mouse macrophages [124]. Despite not understanding how Nek7 acts upstream of NLRP3, these studies provide the first genetic treatment aimed directly at NLRP3. To fully understand the mechanism of activation of NLRP3, future studies on the molecular mechanisms of infection and inflammation are warranted.

By activating transcription of many other cytokines and chemokines, NLRP3 inflammasomes regulate the maturation and release of IL-1, which is essential for host defense against pathogens. As a result, inflammatory diseases like sepsis and acute respiratory distress syndrome (ARDS) are associated with poorer prognoses. Inflammasome activity must be tightly regulated, but the mechanisms that limit excessive activity are unknown. NLRP3 inflammasomes have been regulated by STAMBP, a deubiquitinase STAM-binding protein identified by Joseph S et al. [125] upon contact with Toll-like receptor agonists or bacterial lipopolysaccharide (LPS), STAMBP knockdown by CRISPR/Cas9 gene editing increase the expression of many cytokines and chemokines [126]. It has been discovered that STAMBP knockdown leads directly to an elevated inflammatory response upon TLR activation. STAMBP does not regulate NLRP3 protein abundance, but cellular deubiquitinase depletion increases NLRP3 K63 chain polyubiquitination, which activates NLRP3 inflammasomes. The findings indicate that STAMBP regulates cytokine secretion by nondegradative ubiquitination of NLRP3 that limits excessive inflammasome activation and reduces detrimental IL-1β signaling [127].

Multimolecular signaling apparatus NLRP3 is involved in inflammasome regulation. Guzova et al. [128] developed a simplified, robust, and relevant cell-based model of the NLRP3 inflammasome to overcome the variability among primary immune cells. Human bone marrow mononuclear cells THP1 were used for the study of NLRP3 inflammasomes. THP1 undifferentiated cells express NLRP3 constitutively, and NLRP3 inflammasome activation is initiated by classical NLRP3 activating stimuli, including nigericin, ATP, and urea crystals, resulting in pro-IL-1β cleavage, extracellular release of mature IL-1β, and pyroptosis [129]. As part of CP-456,773's inflammasome selective inhibitor study, potential targets of the inflammasome were investigated using THP1 cells, and the transduction of viral shRNA for gene expression knockdown (KD) was optimized, with KD of NLRP3 itself eliminating inflammasome activation and IL-1β production [130]. THP1 cells lacking ABCB7 or ABCB10 didn't respond to CP-453,773 pharmacology or activate NLRP3 inflammasomes, ruling out these genes as potential targets [131]. THP1 sublines that have been knocked out of ABCb10 using CRISPR/Cas9 were used to confirm the results. The undifferentiated THP1 cells are capable of activating NLRP3 inflammasomes and converting into macrophages without differentiating into them. In a study using undifferentiated THP1 cells to investigate NLRP3 inflammasome biology, the inhibitor CP-453,773 was ruled out as a potential target for ABCb7 and ABCb10.

Post-translational modifications such as ubiquitin control the assembly and activation of inflammasomes. As an immune signaling pathway regulator, DUB counteracts ubiquitin addition. By chemically inhibiting USP7 and USP47, Pablo et al. [132] prevented the formation of inflammasomes by preventing ASC oligomerization and spot formation in macrophages. NLRP3’s ubiquitination status is altered when USP7 and USP47 are inhibited [132]. Moreover, USP7 and USP47 activity increases when inflammasome activator is increased [133]. By depleting USP7 and USP47 in macrophage cells, USP7 and USP47 activation of inflammasomes was reduced. Inflammasome activation and proinflammatory cytokine release can be regulated by USP47/USP7, and two potential therapeutic agents are uncovered by USP7/USP47 inhibitors.

After cytoplasmic recognition of Gram-negative bacteria's cell wall components, including LPS, mouse caspase-11 and its human orthologs caspase-4 and caspase-5 trigger an inflammatory response. The inflammatory response involves pyroptosis and the simultaneous release of IL-1α, as well as the production of IL-1β and IL-18 through the non-classical NLR family, pyrin domain containing 3 (NLRP3) pathway [134]. By utilizing novel gene editing techniques, clustered regularly interspaced short palindromic repeats (CRISPR), and sensitive cellular imaging techniques, we demonstrate that NLRP3 inflammasome is necessary for cytoplasmic LPS-dependent IL-1β production, and its activation is dependent on K + efflux. Il-1β release and pyroptosis are not dependent on NLRP3. As a consequence of these findings, NLRP3 activation and non-classical NLRP3 pathways are both triggered by K + efflux.

PF-04620110, a selective acyl-coa:diacylglycerol acyltransferase-1 (DGAT1) inhibitor, is currently undergoing phase 1 clinical trials for its anti-diabetic properties, which may help regulate chronic inflammation in diabetes type 2 [135]. Through the CRISPR ribonucleoprotein (RNP) system, we deleted mouse DGAT1 with a gRNA targeting DGAT1. Jo et al. [136] showed, however, that PF-04620110 inhibited fatty acid-induced nucleotide-binding domain (FNBD) inflammasome activation in macrophages (NLRP3) by inhibiting NLR. AIM2 and NLR families were not activated by PF-04620110, melanoma 2 was not activated by AIM2, or the NLR family was not activated by AIM2. The inflammasome complex is required for NLRP3 activation, which is inhibited by PF-04620110 [137]. In macrophages, PF-04620110 inhibited NLRP3 inflammasome activation induced by fatty acids. For future clinical trials with or without diabetes, PF-04620110 provides a new direction.

## Summarize

Several chronic inflammation-related diseases, including cancer, are associated with the inflammasome, so its role in clinical practice is receiving increasing attention. In particular, the NLRP3 inflammasome, which contains NACHT, LRR, and PYD domains, is a multimeric, cytoplasmic protein complex that activates to regulate the maturation and secretion of interleukin (IL)-1β (a potent proinflammatory cytokine) and IL-18, affecting metabolism, inflammation, and immunity.

Breast Cancer development has been linked to the NLRP3 inflammasome. A novel diagnostic and therapeutic target for BC may be the NLRP3 inflammasome because of its oncogenic role. The specific roles that they play in TNBC, however, remain unclear. Furthermore, more extensive experimental studies are required to explore its clinical application. Because TNBC occurs early, metastasizes quickly, and has a poor prognosis, treatment is very challenging. TNBC is still primarily treated with chemotherapy, but conventional therapy often fails to work due to resistance to chemotherapy. Inflammatory activation pathways and TNBC appear to have a strong relationship so far. NLRP3 inflammasome should be explored further in different cancer subtypes, which is also the future direction of experimental research.

Inflammasome assembly and pyroptosis cell death are initiated by miRNAs, which regulate gene expression and tumorigenesis. TNBC is heterogeneous and aggressive due to genetic and environmental factors, which has made developing and validating effective targeted strategies difficult. The targeting of miRNAs in combination with anti-tumor drugs has gained wider attention over the past few years because it improves the survival rate of cancer patients by activating NLRP3 in oncogenic pathways. Inflammasomes can be manipulated via microRNAs to improve TNBC treatment, based on the original experiments results.

The role of gene therapy and nanotechnology in cancer treatment is still in its infancy, but it is clear that these technologies will become increasingly important in the years ahead. The overexpression or over-activation of gene products causes cancer cell phenotypes, so it is reasonable to expect that transfection of cells with native p53, CDK inhibitors, NLRP3 inflammasome inhibitors, and other gene products, including synthetic molecules, will suppress these phenotypes. In addition to the advantages of targeted therapy in combination with traditional agents, research in this field and the development of new agents for clinical use are important. Further research is needed for breast cancer and NLRP3 relation despite the many treatments available.

## Data Availability

There is no raw data associated with this review.
